# Anthrax immune globulin improves hemodynamics and survival during *B*. *anthracis* toxin-induced shock in canines receiving titrated fluid and vasopressor support

**DOI:** 10.1186/s40635-017-0159-9

**Published:** 2017-10-23

**Authors:** Dante A. Suffredini, Xizhong Cui, Dharmvir Jaswal, Kenneth E. Remy, Yan Li, Junfeng Sun, Steven B. Solomon, Yvonne Fitz, Mahtab Moayeri, Stephen Leppla, Peter Q. Eichacker

**Affiliations:** 1 Critical Care Medicine Department, Clinical Center, National Institutes of Health, Bldg 10, Rm 2C145, Bethesda, MD 20892 USA; 2 Division of Critical Care Medicine, Department of Pediatrics, Washington University School of Medicine, St. Louis, MO 63110 USA; 3 National Institute of Allergy and Infectious Disease, National Institutes of Health, Bethesda, MD 20892 USA

**Keywords:** *B. anthracis*, Anthrax infection, Anthrax immune globulin, Sepsis, Lethal toxin, Edema toxin

## Abstract

**Background:**

Although anthrax immune globulin (AIG) improved survival in antibiotic-treated Bacillus anthracis-challenged animal models, whether it adds to the benefit of conventional hemodynamic support for *B. anthracis* toxin-associated shock is unknown.

**Methods:**

We therefore tested AIG in sedated, mechanically ventilated canines challenged with 24-h B. anthracis lethal and edema toxin infusions and supported for 96 h with a previously demonstrated protective regimen of titrated normal saline and norepinephrine.

**Results:**

Compared to controls, proportional survival (%) was increased with AIG treatment started 4 h before (33 vs. 100%, *n* = 6 each) or 2 h (17 vs. 86%, *n* = 6 and 7 respectively) or 5 h (0 vs. 67%, *n* = 3 each) after the start of toxin (*p* ≤ 0.05) and overall [3 survivors of 15 controls (20%) vs. 14 of 16 AIG animals (88%); *p* = 0.006]. Averaged across treatment times, AIG increased blood pressure at 48 h and decreased norepinephrine requirements at 72 h (*p* ≤ 0.02), increased left ventricular ejection fraction at 48 and 72 h (*p* ≤ 0.02), and increased urine output and decreased net fluid balance at 72 and 96 h (*p* ≤ 0.04). AIG also reduced acidosis and renal and hepatic injury markers between 24 and 96 h.

**Conclusions:**

These findings further support AIG’s potential benefit for patients with *B*. *anthracis* infection and developing toxin-associated shock.

**Electronic supplementary material:**

The online version of this article (10.1186/s40635-017-0159-9) contains supplementary material, which is available to authorized users.

## Background

*Bacillus anthracis* (anthrax) lethal and edema toxins (LT and ET respectively) are strongly linked to the pathogenesis of shock during anthrax infection. The Centers for Disease Control and Prevention (CDC) Guidelines now recommend that, besides antibiotic and cardiopulmonary support, patients with systemic anthrax infection receive an anti-toxin agent [1–7]. At this time, anthrax immune globulin (AIG; Emergent BioSolutions, Gaithersburg, MD) is the only anti-toxin agent with US Food and Drug Administration (FDA) approval that has also been administered to patients with anthrax infection [7, 8]. Anthrax immune globulin is a polyclonal antibody directed at the protective antigen (PA) component of the toxins which was originally produced by Cangene Corp. until this company was acquired by Emergent BioSolutions [9, 10].

Due to the infrequency of invasive anthrax infection in humans, AIG received approval based on studies showing it increased survival in an anthrax-challenged and antibiotic-treated rabbit model [11]. However, since these studies were conducted at high biosafety levels limiting animal contact, they did not determine whether AIG would improve outcome when combined with the titrated hemodynamic support patients with infection- and toxin-related shock receive. Experiences with AIG during systemic anthrax infection in 3 isolated inhalational cases and 15 cases from an outbreak due to contaminated heroin injection have also not answered this question [12–15]. In fact, in this outbreak, AIG did not improve outcome in the 15 patients receiving treatment compared to 28 non-recipients, although recipients were sicker [12]. Determining whether AIG adds to the beneficial effects of hemodynamic support would provide additional insight and confidence as to its clinical efficacy.

We previously developed a 96-h sedated, instrumented, and mechanically ventilated canine model of anthrax toxin-associated shock in which LT and ET were infused over 24 h to simulate release during infection [4]. In this model, support with fluids and vasopressors titrated with pulmonary and systemic arterial catheters improved hemodynamics and survival during the development of toxin-associated shock [16]. Therefore, to further explore AIG’s efficacy, here we compared its effects to placebo when administered earlier or later in canines challenged with 24-h infusions of LT and ET and treated with this protective regimen of hemodynamic support.

## Methods

### Study design

**Fig. 1:**
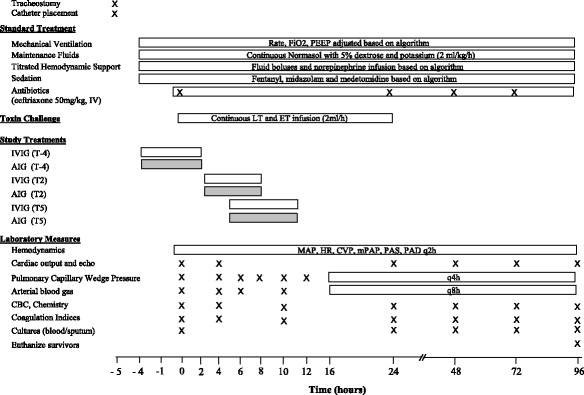
Timeline of experimental interventions, measurements, and treatments as described in the “Methods” section. At time 0 (T0), all animals were started on a continuous 24-h infusion of lethal toxin (LT) and edema toxin (ET) together. The animals were randomly assigned to receive intravenous immune globulin (IVIG) as control or anthrax immune globulin (AIG) at T-4 or T2 in six weekly experiments, at T2 or T5 in one experiment, and at T5 in one experiment. To prevent hypersensitivity reactions in the animals, infusions of AIG or IVIG were administered in gradually increasing concentrations such that the first 50% of the total dose was given over 4 h and then the second 50% over 2 h and 20 min, starting from the designated treatment time (see Additional file 1: Table S1). Hemodynamic support included a single bolus of 20 mL/kg of normal saline if the pulmonary capillary wedge pressure (PCWP, checked every 2 h for the first 8 h and every 4 h thereafter) was < 10 mmHg. Also, if at any time the mean arterial pressure (MAP) decreased to < 80 mmHg for > 5 min, a norepinephrine (NE) infusion was initiated at 0.2 μg/kg/min and if necessary increased in stepwise fashion every 5 min to 0.6, 1, or a maximum of 2 μg/kg/min. NE was titrated down in a stepwise fashion if MAP was > 100 mmHg for 5 min. Abbreviations: ABG, arterial blood gas; CBC, complete blood count; CVP, central venous pressure; HR, heart rate; LVEF, left ventricular ejection fraction (measured with echocardiography); PAS and PAD, pulmonary systolic and diastolic pressures respectively

Four purpose-bred beagles (9–12 kg) with central venous, pulmonary arterial, systemic arterial, and urinary catheters and tracheostomy tubes were studied weekly over 8 weeks (*n* = 32). Using standardized ICU protocols, sedation, fluids, vasopressors, and mechanical ventilation were applied similarly across all groups (see below) [4, 16]. Animals were anesthetized, mechanically ventilated, and had a tracheostomy and catheters placed (Fig. 1). Then, after changing from anesthesia to continuous sedation and analgesia and starting at time 0 h (T0), all animals were challenged with a 24-h infusion of LT and ET and treated with titrated hemodynamic support (see below). In six initial experiments, the animals were assigned blindly to one of four treatment groups based on the type and designated time of treatment: (1) intravenous human immunoglobulin (IVIG) initiated 4 h before the start of toxin (T-4 control; *n* = 6); (2) IVIG initiated 2 h after the start of toxin (T2 control; *n* = 6); (3) AIG initiated 4 h before the start of toxin (T-4 AIG; *n* = 6); or (4) AIG initiated 2 h after the start of toxin (T2 AIG; *n* = 6) (Additional file 1: Table S1, Fig. 1). To explore whether treatment started later than T2 would also show benefit, two subsequent experiments were performed. Animals received IVIG or AIG initiated at 2 h (T2 control and T2 AIG; *n* = 1 animal with each treatment) or 5 h (T5 control and T5 AIG; *n* = 1 animal with each treatment) in one experiment or IVIG or AIG initiated at 5 h only in one experiment (T5 control and T5 AIG; *n* = 2 with each treatment). One control received diluent only and not IVIG at the T2 time point and was not included in analysis. Thus, the total number of animals starting in each group was as follows: IVIG at T-2, *n* = 6; AIG at T-2, *n* = 6; IVIG at T2, *n* = 6; AIG at T2, *n* = 7; IVIG at T5, *n* = 3; and AIG at T5, *n* = 3. Cardiopulmonary and other laboratory measures were performed prior to the initiation of toxin and continued until the end of the study (96 h), at regular intervals as outlined in Fig. 1 [4, 16].

### Toxin and treatments

Lethal and edema toxin infusions were prepared and administered as described previously [4]. AIG was supplied by the CDC (lot number 10602912, Cangene Corporation, Winnipeg, Canada) at a standard concentration of 2.18 mg/mL in 50-mL vials. AIG animals received the manufacturer-recommended pediatric dose (1.5 vials for a 10–20-kg patient) [17]. Control animals received an equivalent dose of IVIG (Gamunex, Grifols Therapeutics, Clayton, NC). To minimize hypersensitivity reactions to the human protein, AIG and IVIG were administered in gradually escalating doses as outlined in Additional file 2: Table S2. The animals received the initial 50% of their treatment dose over 4 h before and the final 50% over 2 h after the designated treatment times (Fig. 1). The animals also received diphenhydramine (1 mg/kg IV) every 6 h for three doses and famotidine (1 mg/kg) every 12 h starting with the AIG and IVIG treatments.

Prior to AIG or IVIG, animals received up to three boluses (20 cm^3^/kg) of saline until a pulmonary capillary wedge pressure (PCWP) of at least 10 mmHg was achieved. Thereafter, animals with a PCWP < 10 mmHg after routine measurements were treated with additional 20 mL/kg normal saline boluses (Fig. 1). Any time the mean arterial blood pressure (MAP) was < 80 mmHg for > 5 min, a norepinephrine infusion was started at 0.2 μg/kg/min and increased stepwise to a maximum of 2 μg/kg/min if needed. Norepinephrine was similarly decreased stepwise if MAP was > 110 mmHg. Administered norepinephrine amounts were recorded every 2 h for each animal. Ventilator management, temperature control, and sedation with midazolam, fentanyl, and medetomidine were managed uniformly for all animals based on previously reported protocols [18]. Additional animal care included pharmacologic prophylaxis for gastrointestinal stress ulcers (famotidine), deep vein thrombosis (subcutaneous heparin), and ceftriaxone to prevent catheter-related infection [18]. Animal technicians administering support to animals were blinded throughout the study to treatment assignments (i.e., AIG vs. IVIG).

### Statistics

Survival times were plotted in Kaplan-Meyer curves and analyzed using stratified log-rank test and stratified Cox proportional hazard model. For all other variables, changes from baseline values at each subsequent time point were analyzed. To evaluate shock reversal, we standardized MAP and NE using *Z*-scores and then calculated a Shock Index score based on the difference of the MAP *Z*-score and NE *Z*-score. Increased or decreased scores represent improved or worsened hemodynamic function respectfully. Linear mixed models were used to account for the pairing of animals within each cycle. Standard residual diagnostics were used to check model assumptions. Logarithm-transformation was used when necessary. SAS version 9.3 (Cary, NC) was used for all analyses. Data is presented from the completion of toxin challenge at 24 h to the end of the study at 96 h. All *p* values are two-tailed and considered significant if *p* ≤ 0.05. One animal in the T2 control group received D5W and not IVIG as placebo and was not included in the analysis.

## Results

### Survival

**Fig. 2:**
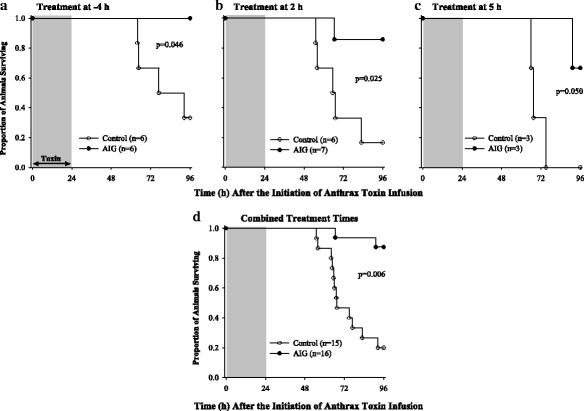
Comparison of the survival times in animals treated with anthrax immune globulin (AIG, closed circle) or intravenous immune globulin (control, open circle) administered 4 h before (**a**) or 2 h (**b**) or 5 h (**c**) after the start of a 24-h infusion of lethal and edema toxins together. Panel **d** shows the survival analysis after combining data from the three individual treatment times. The numbers of animals studied and the *p* values for comparing the control and AIG groups are shown in each panel

Six initial experiments showed that treatment with AIG started at T-4 and T2 treatment times had similar, beneficial effects on survival (Additional file 1: Table S1). To explore whether treatment started later than T2 would also show benefit, it was decided after completion of the first six experiments that two final ones would be conducted. These experiments tested treatment started at T2 and T5 in one experiment and only T5 in another (see the “Methods” section). Limited animal resources prevented further study at this later time point. Overall, in these animals challenged with 24-h infusions of anthrax LT and ET and administered titrated cardiopulmonary support, compared to IVIG (controls), AIG treatment increased survival at 96 h whether it was initiated 4 h before the start of toxin [2 survivors of 6 total controls (33%) vs. 6 survivors of 6 total AIG-treated animals (100%), *p* = 0.046] or 2 or 5 h after the start of toxin [1 of 6 (17%) vs. 6 of 7 (86%), *p* = 0.025 at 2 h and 0 of 3 (0%) vs. 2 of 3 (66%), *p* = 0.050 at 5 h] (Fig. 2). The survival effects of AIG did not differ across the three treatment times (*p* = ns), and when combined, AIG increased overall survival highly significantly [3 survivors of 15 controls (20%) vs. 14 survivors of 16 AIG animals (88%), *p* = 0.006]. Since AIG had similar survival effects comparing treatment times, subsequent analysis combined data from the three times to increase the power to identify a basis for AIG survival benefit. For further reference, Additional files 3, 4, 5, 6, and 7: Tables S3 to S7 show the mean (± SEM) differences in the effects of AIG treatment comparing treatment started at T2 or T5 versus treatment started at T-4 for each of the parameters presented below and at each time point of the study. Also shown are the levels of significance for each of those differences.

### Hemodynamic and pulmonary function

At baseline before toxin infusion, only 1 out of 40 parameters had a significant *p* value comparing control and AIG and this was no more than what would be expected by chance alone (Additional file 8: Table S8). Data is therefore shown as serial mean (± SEM) changes from baseline for all parameters except fluid and urine amounts, which are shown as hourly amounts averaged for each preceding 24-h period (see below).

**Fig. 3:**
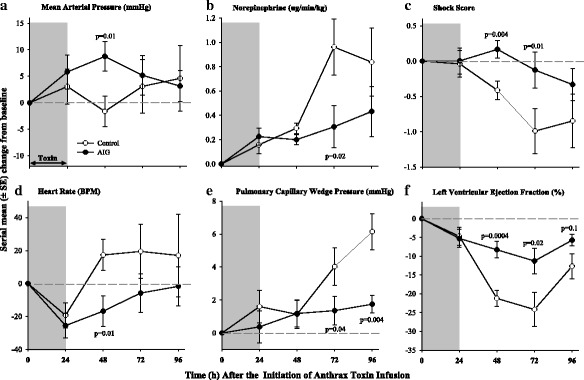
Serial mean (± SEM) changes from baseline in mean arterial pressure (**a**), norepinephrine infusion rate (**b**), shock score (**c**), heart rate (**d**), pulmonary capillary wedge pressure (**e**), and left ventricular ejection fraction (**f**) comparing animals receiving anthrax immune globulin (AIG, black circles) versus intravenous immune globulin (control, open circles). Levels of significance (*p* values) for time points at which the groups differed significantly are provided in the figure. The number of animals studied at the 24-, 48-, 72-, and 96-h time points for the control group was 15, 15, 7, and 3, respectively, and for the AIG group was 16, 16, 15, and 14 respectively

**Fig. 4:**
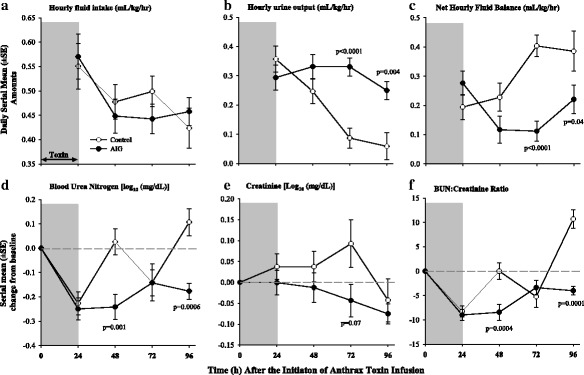
Serial mean (± SEM) hourly fluid intakes (**a**), urine outputs (**b**), and net fluid balances (**c**) for preceding 24-h periods and serial mean (± SEM) changes from baseline in blood urea nitrogen (BUN) (**d**), creatinine (**e**), and BUN to creatinine ratio (**f**) comparing animals receiving anthrax immune globulin (AIG, black circles) versus intravenous immune globulin (control, open circles). Levels of significance (*p* values) for time points at which the groups differed significantly are provided in the figure. The number of animals studied at the 24-, 48-, 72-, and 96-h time points for the control group was 15, 15, 7, and 3, respectively, and for the AIG group was 16, 16, 15, and 14 respectively

**Fig. 5:**
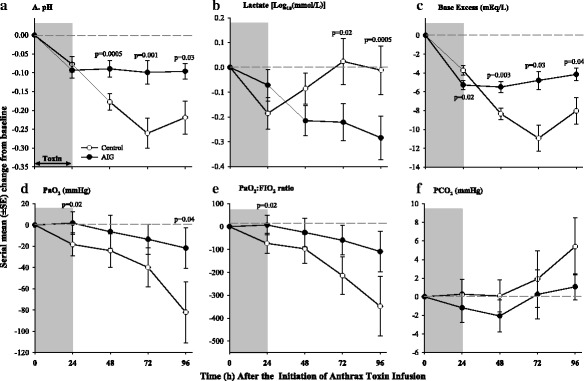
Serial mean (± SEM) changes from baseline in pH (**a**), lactate (**b**), base excess (**c**), arterial oxygen pressure (PaO_2_) (**d**), PaO_2_ to fractional inspired oxygen concentration (PaO_2_:FiO_2_) (**e**), and arterial carbon dioxide pressure (PaCO_2_) (**f**) comparing animals receiving anthrax immune globulin (AIG, black circles) versus intravenous immune globulin (control, open circles). Levels of significance (*p* values) for time points at which the groups differed significantly are provided in the figure. The number of animals studied at the 24-, 48-, 72-, and 96-h time points for the control group was 15, 15, 7, and 3, respectively, and for the AIG group was 16, 16, 15, and 14 respectively

Compared to controls, AIG increased mean arterial blood pressures (MAPs) at 48 h and decreased heart rate and norepinephrine requirements at 72 h respectively (*p* ≤ 0.02) (Fig. 3). From 48 to 96 h, AIG increased the shock index, and these improvements were significant at 48 and 72 h (*p* ≤ 0.01). Hourly fluid intake was greatest during toxin infusion but did not differ between treatments. However, compared to controls, AIG increased urine output and decreased net fluid balance at 72 and 96 h (*p* ≤ 0.04) (Fig. 4). Consistent with the reduced fluid balance with AIG, PCWP, which reflects left ventricular filling pressure, was also decreased at 72 and 96 h (*p* ≤ 0.04). Despite decreased PCWP, AIG improved cardiac function as reflected by increases in left ventricular ejection fraction at 48, 72, and 96 h (*p* = 0.0004, 0.02, and 0.10 respectively). AIG also increased stroke volume index and left ventricular stroke work index at 48 and 72 h (*p* ≤ 0.01 for each, data not shown). AIG increased systemic vascular resistance and decreased pulmonary artery pressure at 72 h (*p* ≤ 0.02) and did not alter central venous pressure or cardiac index significantly at any time point (Additional file 9: Figure S1). AIG increased pH and base excess at 48, 72, and 96 h and decreased lactate at 72 and 96 h (*p* ≤ 0.03) (Fig. 5). AIG increased arterial oxygen pressure (PaO_2_) at 24 and 96 h (*p* ≤ 0.04) and increased PaO_2_/FiO_2_ significantly or in a significant trend at 24, 72, and 96 h (*p* = 0.02, *p* = 0.08, and *p* = 0.06 respectfully) (Fig. 5).

### Renal, hepatic, and hematologic data

**Fig. 6:**
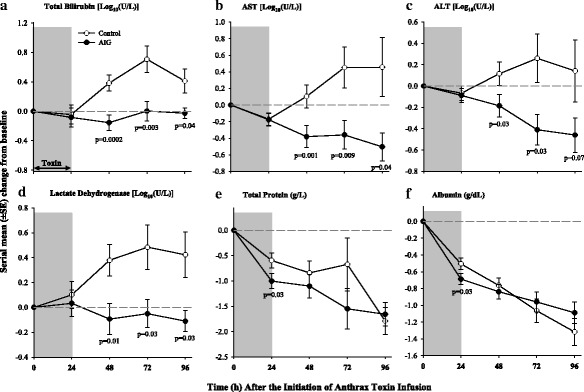
Serial mean (± SEM) changes from baseline in total bilirubin (**a**), aspartate aminotransferase (AST) (**b**), alanine aminotransferase (ALT) (**c**), lactate dehydrogenase (LDH) (**d**), total protein (**e**), and albumin (**f**) comparing animals receiving anthrax immune globulin (AIG, black circles) versus intravenous immune globulin (control, open circles). Levels of significance (*p* values) for time points at which the groups differed significantly are provided in the figure. The number of animals studied at the 24-, 48-, 72-, and 96-h time points for the control group was 15, 15, 7, and 3, respectively, and for the AIG group was 16, 16, 15, and 14 respectively

**Fig. 7:**
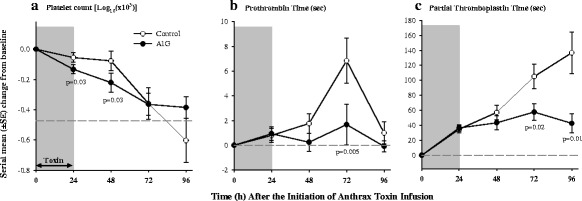
Serial mean (± SEM) changes from baseline in platelet count (**a**), prothrombin time (**b**), and partial thromboplastin time (**c**) comparing animals receiving anthrax immune globulin (AIG, black circles) versus intravenous immune globulin (control, open circles). Levels of significance (*p* values) for time points at which the groups differed significantly are provided in the figure. The number of animals studied at the 24-, 48-, 72-, and 96-h time points for the control group was 15, 15, 7, and 3, respectively, and for the AIG group was 16, 16, 15, and 14 respectively

Compared to controls, AIG decreased BUN at 48 and 96 h, creatinine at 72 h, and the BUN to creatinine ratio at 48 and 96 h (*p* ≤ 0.001 except for creatinine which was *p* = 0.07) (Fig. 4). AIG reduced serum potassium levels at 48, 72, and 96 h (*p* ≤ 0.02) and increased serum bicarbonate at 72 h (*p* = 0.02) (data not shown). AIG decreased bilirubin and aspartate and alanine aminotransferases (AST and ALT respectively), and lactate dehydrogenase (LDH) at 48, 72, and 96 h (all *p* ≤ 0.04 except ALT at 96 h which was *p* = 0.07) (Fig. 6). AIG also decreased prothrombin time at 72 h and partial thromboplastin time at 72 and 96 h (*p* ≤ 0.02) (Fig. 7). AIG increased serum calcium at 48 and 72 h (*p* < 0.03) and decreased total protein and albumin at 24 h (*p* ≤ 0.03, Fig. 6). Finally, AIG decreased platelets at 24 and 48 h (*p* = 0.03, Fig. 7) but did not alter white blood cell counts or hemoglobin levels (*p* = ns).

## Discussion

In canines challenged with 24-h LT and ET infusions and provided a protective regimen of titrated hemodynamic support similar to what is applied clinically for shock, AIG treatment improved survival. This protection was evident even when the AIG dose was administered from 5 to 11 h after the start of toxin. These findings add to data from an antibiotic-treated, anthrax-challenged animal model, suggesting that AIG will have benefit when administered in patients with systemic infection and developing toxin-related shock and organ injury [19].

Based on evolving concepts regarding LT and ETs’ pathogenic effects, AIG may have improved survival several ways. First, both LT and ET appear to cause endothelial injury, which in vivo could produce extravasation and loss of intravascular volume [5, 20–22]. This fluid movement can produce either systemic hypotension with secondary reductions in organ perfusion or direct organ injury if tissue edema interferes with oxygen and nutrient uptake [23–27]. Although control and AIG animals received similar larger supporting fluid volumes over the first 24 h, subsequent urine output was increased and net fluid balance decreased with AIG, suggesting that treatment prevented extravasation of fluid. Consistent with this, total protein, albumin, and platelets were decreased early or later with AIG, suggesting that retention of intravascular volume caused hemodilution. Retained intravascular volume with AIG may have helped improve blood pressure and reduced vasopressor needs. Second, studies now show that ET reduces arterial contractile force and response to catecholamines, resulting in increased arterial relaxation [28, 29]. Lethal toxin may also alter vascular smooth muscle function [30]. These effects in vivo would cause arterial dilation and hypotension. Thus, AIG may have helped maintain arterial contractile function and its response to endogenous catecholamines and norepinephrine treatment, effects consistent with the increased blood pressure and reduced norepinephrine requirements in treated animals. Third, LT has been associated in several animal models including this canine one, with left ventricular systolic dysfunction [6, 30–32]. It is therefore noteworthy that AIG produced increased left ventricular ejection fraction and stroke volume from 48 to 96 h. Although this effect may have been related to improved preload due to retention of intravascular volume, PCWP was actually reduced with AIG.

Overall, whether related to improved intravascular volume or arterial contractile or myocardial systolic function, increased survival with AIG likely resulted from increased organ perfusion and reduced organ injury. Treated animals had decreased lactate and improved lung, kidney, and liver function as reflected by improved oxygenation and reductions in creatinine, BUN, liver enzymes, and clotting times.

At this time, besides AIG, Raxibacumab (GlaxoSmithKline, Rockville, MD) is the other anti-toxin agent that has received FDA approval for use during anthrax infection and been included in the SNS. Raxibacumab is a humanized chimpanzee monoclonal antibody (mAb) directed against PA [4, 33–35]. This agent also received FDA approval based on a study showing it improved survival in anthrax-challenged and antibiotic-treated rabbits [36]. Although Raxibacumab has not been administered clinically, we previously tested it in our toxin-challenged canine model [16, 37]. All animals in that prior study received the same titrated hemodynamic and ventilatory support as the animals in the present study received. Raxibacumab infused over 30 min increased survival when administered at the time of or at 9 h after the start of toxin challenge. It also increased blood pressure and decreased norepinephrine requirements and improved urine output, fluid clearance, and liver function. Thus, based on data from both bacteria-challenged models treated with antibiotics and our toxin-challenged canine model treated with titrated cardiopulmonary support, AIG and Raxibacumab appear to have comparable beneficial effects. However, there are potential advantages when comparing polyclonal to monoclonal preparations [38]. A polyclonal preparation is directed at multiple epitopes and might be less likely to lose effectiveness in the setting of a bacterial strain engineered to produce a toxin resistant to available monoclonal preparations. Also, a polyclonal preparation can also elicit several different effector cell functions which may improve ultimate bacterial clearance. Although mixes of monoclonal antibodies can be prepared to address this problem, that can be a costly solution. To fully determine these agents’ similarity will require an analysis of their effects when applied in patients with similarly severe bacterial infection. A humanized mouse anti-PA mAb, ETI-204, has also received FDA approval based on studies in anthrax-challenged and antibiotic-treated animal models but has not been tested in this toxin-challenged canine model [39–41].

Prior measurements in this canine model suggest that the toxin challenges produced circulating toxin levels comparable to ones with anthrax infection. Previously, for canines challenged with 24-h LT infusions at doses similar to the ones used here, PA levels (mean ± SEM) at toxin infusion completion were 34 ± 3 ng/mL. This level was within the range of ones measured in cynomolgus monkeys challenged with LD200 doses of anthrax spores, in which PA levels increased from 1.3 ± 2.3 ng/mL at 30 h to 178.3 ± 37.4 ng/mL at 72 h [4]. The level of 34 ± 3 ng/mL was also similar to one of 68.7 ng/mL reported for a patient with inhalational anthrax infection [42].

The latest treatment tested in this study that was started and completed 5 and 11 h, respectively, following the initiation of toxin challenge had beneficial effects comparable to earlier treatment times. However, even this treatment is much earlier than will likely occur in patients presenting during an actual outbreak of infection. In the 2009 outbreak of soft tissue infection, the median time (IQR) from the onset of symptoms to hospital presentation was 2 days [1, 5], and administration of AIG occurred hours after that related to the need to confirm the presence *B*. *anthracis* [12]. Administration of AIG in isolated cases of inhalational disease have also occurred well after the onset of symptoms. Whether AIG or other antibody preparations will prove beneficial with these types of delay is unclear. Two studies in rabbits did show that initiation of either AIG or Raxibacumab along with antibiotics up to 96 h after challenge with live *B*. *anthracis* resulted in trends towards improved survival compared to antibiotics alone [19, 33].

Due to the sensitivity of canines to the human protein in AIG, treatment had to be administered in escalating doses over a 6-h and 22-min period. This regimen resulted in half of each effective dose being given prior to and the other half following the designated treatment time point. However, this regimen was similar to the one employed during AIG administration in the outbreak of anthrax soft tissue infection in the UK [12]. Notably in the present study, the T2 and T5 groups began to receive AIG treatment as animals were beginning to require fluid and vasopressor support for developing hypotension.

A smaller number of animals were studied with AIG at the T9-designated treatment time point than at earlier ones. However, AIG treatment at this later time point did have a significant effect on survival that was similar to that of earlier treatment, and the use of additional limited animal resources for study with the T5 treatment was not justified.

## Conclusions

Findings in this canine model demonstrate that AIG treatment can improve survival when combined with conventional hemodynamic support in anthrax toxin-challenged animals. Prior studies by others have shown that AIG can improve survival when combined with antibiotic therapy in anthrax-challenged animal models. Together these findings provide support for the use of AIG in patients with systemic anthrax infection. Other similar findings would also support the use of Raxibacumab. However, until these anti-toxin agents are investigated along with both antibiotic and cardiopulmonary support either in anthrax-challenged animal models or in infected patients, their actual effectiveness will be unknown.

## Additional files

Additional file 1: Table S1. Survival times (h) for animals assigned to receive anthrax immune globulin (AIG) or intravenous immune globulin (control) treatment starting 4 h before (T-4) or 2 h (T2) or 5 h (T5) after the start of a 24-h *B*. *anthracis* toxin infusion in eight experiments. (DOCX 12 kb)

Additional file 2: Table S2. Desensitization protocol for anthrax immune globulin and intravenous immunoglobulin (control) showing the time and rate at which each escalating dilution of the treatments was administered. (DOCX 11 kb)

Additional file 3: Table S3. Differences in the effects of treatment at T2 or T5 versus T0 for hemodynamic parameters. (DOCX 13 kb)

Additional file 4: Table S4. Differences in the effects of treatment at T2 or T5 versus T0 for fluid balance and renal function parameters. (DOCX 13 kb)

Additional file 5: Table S5. Differences in the effects of treatment at T2 or T5 versus T0 for arterial blood gas parameters. (DOCX 14 kb)

Additional file 6: Table S6. Differences in the effects of treatment at T2 or T5 versus T0 for liver function parameters. (DOCX 14 kb)

Additional file 7: Table S7. Differences in the effects of treatment at T2 or T5 versus T0 for platelets and coagulation parameters. (DOCX 13 kb)

Additional file 8: Table S8. Mean (± SEM) values for parameters at baseline immediately before the start of toxin infusion in animals treated with anthrax immune globulin (AIG) or intravenous immune globulin (control) and the level of significance (*p* value) for the comparison of each parameter in the two groups. (DOCX 18 kb)

Additional file 9: Figure S1. Serial mean (± SEM) changes from baseline in systeic vascular resistance index (a), pulmonary arterial pressure (b), central venous pressure (c) and cardiac index (d) comparing animals receiving anthrax immune globulin (AIG, black circles) versus intravenous immune globulin (control, open circles). Levels of significance (p values) for time points at which the groups differed significantly are provided in the figure. (PDF 14 kb)
