# Supplementary material for: Anthrax immune globulin improves hemodynamics and survival during B. anthracis toxin-induced shock in canines receiving titrated fluid and vasopressor support
Source: Intensive Care Med Exp. 2017 Oct 23;5:48. doi: 10.1186/s40635-017-0159-9 (PMC5651533; doi:10.1186/s40635-017-0159-9)
Supplement: Supplementary file 1 — Survival times (h) for animals assigned to receive anthrax immune globulin (AIG) or intravenous immune globulin (control) treatment starting 4 h before (T-4) or 2 h (T2) or 5 h (T5) after the start of a 24-h B. anthracis toxin infusion in eight experiments. (DOCX 12 kb) [file 40635_2017_159_MOESM1_ESM.docx]

| Additional file 1: Table S1. Survival times (h) for animals assigned to receive anthrax immune globulin (AIG) or intravenous immune globulin (control) treatment starting four hours before (T-4) or 2h (T2) or 5h (T5) after the start of a 24h *B. anthracis* toxin infusion in eight experiments* | | | | | | |
| --- | --- | --- | --- | --- | --- | --- |
| Experiment  Number | Control  T-4 | AIG  T-4 | Control  T2 | AIG  T2 | Control  T5 | AIG  T5 |
| 1 | 77 | 96 | 48** | 66.5 | 1 | 77 |
| 2 | 64.6 | 96 | 55 | 96 |  |  |
| 3 | 96 | 96 | 96 | 96 |  |  |
| 4 | 96 | 96 | 67.1 | 96 |  |  |
| 5 | 92.5 | 96 | 56 | 96 |  |  |
| 6 | 64 | 96 | 56 | 96 |  |  |
| 7 |  |  | 65.5 | 96 | 67.5 | 91 |
| 8 |  |  |  |  | 75/66 | 96/96 |
| * As described in the methods, 50% of each AIG and control treatment was administered over 4h before and 50% was administered over 2h and 20min after the designated treatment times; **This animal received D5W as control treatment and is not included in analysis | | | | | | |
