# Supplementary material for: Anthrax immune globulin improves hemodynamics and survival during B. anthracis toxin-induced shock in canines receiving titrated fluid and vasopressor support
Source: Intensive Care Med Exp. 2017 Oct 23;5:48. doi: 10.1186/s40635-017-0159-9 (PMC5651533; doi:10.1186/s40635-017-0159-9)
Supplement: Supplementary file 2 — Desensitization protocol for anthrax immune globulin and intravenous immunoglobulin (control) showing the time and rate at which each escalating dilution of the treatments was administered. (DOCX 11 kb) [file 40635_2017_159_MOESM2_ESM.docx]

| Additional file 2: Table S2. Desensitization protocol for anthrax immune globulin and intravenous immunoglobulin (control) showing the time and rate at which each escalating dilution of the treatments was administered | | |
| --- | --- | --- |
| Time (min) | Dilution | Rate (ml/h) |
| 0-20 | 1:50 | 0.12 |
| 20-40 | 1:25 | 0.24 |
| 40-60 | 1:10 | 0.6 |
| 60-80 | 1:5 | 1.2 |
| 80-100 | 1:3 | 1.8 |
| 100-120 | 1:2 | 3 |
| 120-140 | 1:1 | 6 |
| 140-160 | 0 | 12 |
| 160-382 | 0 | 18 |
