# Supplementary material for: Anthrax immune globulin improves hemodynamics and survival during B. anthracis toxin-induced shock in canines receiving titrated fluid and vasopressor support
Source: Intensive Care Med Exp. 2017 Oct 23;5:48. doi: 10.1186/s40635-017-0159-9 (PMC5651533; doi:10.1186/s40635-017-0159-9)
Supplement: Supplementary file 3 — Differences in the effects of treatment at T2 or T5 versus T0 for hemodynamic parameters. (DOCX 13 kb) [file 40635_2017_159_MOESM3_ESM.docx]

| Additional file 3: Table S3. Differences in the effects of treatment at T2 or T5 versus T0 for hemodynamic parameters | | | | | | | | | |
| --- | --- | --- | --- | --- | --- | --- | --- | --- | --- |
| Parameter  (Unit) | Differences in the effect of treatment at  T2 versus T-4 (p-value) | | | |  | Differences in the effect of treatment at  T5 versus T-4 (p-value) | | | |
|  | Time of measurement | | | |  | Time of measurement | | | |
|  | 24 | 48 | 72 | 96 |  | 24 | 48 | 72 | 96 |
| MAP  (mmHg) | 7±9  (0.46) | -14±9  (0.12) | -12±10  (0.28) | -3±15  (0.87) |  | 17±11  (0.15) | -11±11  (0.33) | 14±12  (0.27) | - |
| NE  (ug/kg/min) | 0.08±0.17  (0.66) | -0.11±0.12  (0.38) | 0.62±0.58  (0.32) | 0.89±0.30  (0.02) |  | 0.20±0.21  (0.35) | 0.07±0.15  (0.67) | -0.55±0.74  (0.48) | - |
| Shock Score | 0.22±0.46  (0.65) | -0.51±0.40  (0.22) | -1.20±0.49  (0.04) | -1.19±0.58  (0.09) |  | 0.54±0.58  (0.36) | -0.56±0.50  (0.27) | 1.17±0.60  (0.08) | - |
| Heart Rate  (BPM) | 28±22  (0.22) | 19±29  (0.52) | 6±46  (0.91) | 23±60  (0.72) |  | 35±27  (0.22) | 3±37  (0.93) | 45±59  (0.47) | - |
| PCWP  (mmHg) | 2±1  (0.20) | 1±2  (0.57) | -2±2  (0.28) | 1±3  (0.76) |  | 9±2  (<0.0001) | 8±2  (p=0.001) | 1±2  (0.55) | - |
| LVEF  (%) | -5±6  (0.43) | 10±7  (0.17) | 2±12  (0.85) | 3±9  (0.74) |  | -4±8  (0.63) | 9±9  (0.34) | 14±14  (0.37) | - |
| MAP – mean arterial blood pressure; NE – norepinephrine; PCWP – pulmonary capillary wedge pressure; LVEF – left ejection fraction | | | | | | | | | |
