# Supplementary material for: Anthrax immune globulin improves hemodynamics and survival during B. anthracis toxin-induced shock in canines receiving titrated fluid and vasopressor support
Source: Intensive Care Med Exp. 2017 Oct 23;5:48. doi: 10.1186/s40635-017-0159-9 (PMC5651533; doi:10.1186/s40635-017-0159-9)
Supplement: Supplementary file 4 — Differences in the effects of treatment at T2 or T5 versus T0 for fluid balance and renal function parameters. (DOCX 13 kb) [file 40635_2017_159_MOESM4_ESM.docx]

| Additional file 4: Table S4. Differences in the effects of treatment at T2 or T5 versus T0 for fluid balance and renal function parameters | | | | | | | | | |
| --- | --- | --- | --- | --- | --- | --- | --- | --- | --- |
| Parameter  (Unit) | Differences in the effect of treatment at  T2 versus T-4 (p-value) | | | |  | Differences in the effect of treatment at  T5 versus T-4 (p-value) | | | |
|  | Time of measurement | | | |  | Time of measurement | | | |
|  | 24 | 48 | 72 | 96 |  | 24 | 48 | 72 | 96 |
| Fluid intake  (mL/kg/h) | -0.01±0.11  (0.89) | 0.12±0.09  (0.19) | 0.05±0.09  (0.56) | 0.20±0.12  (0.12) |  | 0.09±0.14  (0.53) | 0.10±0.11  (0.41) | 0.01±0.11  (0.94) | - |
| Fluid output  (mL/kg/h) | 0.08±0.12  (0.49) | 0.10±0.14  (0.46) | -0.11±0.09  (0.26) | -0.05±0.12  (0.67) |  | -0.01±0.15  (0.96) | 0.01±0.17  (0.98) | -0.09±0.12  (0.49) | - |
| Net fluid balance  (mL/kg/h) | -0.10±0.12  (0.40) | 0.03±0.16  (0.88) | 0.19±0.11  (0.10) | 0.22±0.16  (0.20) |  | 0.10±0.14  (0.52) | 0.09±0.20  (0.65) | 0.10±0.14  (0.48) | - |
| BUN  [log_10_ (mg/dL)] | -0.53±0.15  (0.37) | -0.25±0.15  (0.10) | 0.34±0.15  (0.05) | -0.13±0.08  (0.16) |  | 0.09±0.18  (0.62) | -0.34±0.18  (0.08) | 0.78±0.19  (0.003) | - |
| Creatinine  [log_10_ (mg/dL)] | -0.003±0.1  (0.97) | -0.22±0.11  (0.05) | 0.11±0.12  (0.39) | -0.15±0.10  (0.17) |  | -0.01±0.11  (0.97) | -0.11±0.13  (0.97) | -0.54±0.15  (0.006) | - |
| BUN/Cre | -4.2±3.6  (0.26) | -2.5±4.2  (0.56) | 8.1±5.3  (0.16) | 2.7±4.8  (0.59) |  | -0.23±4.4  (0.90) | -13.5±5.1  (0.02) | 20.9±6.7  (0.01) | - |
| Bun – blood urea nitrogen | | | | | | | | | |
