# Supplementary material for: Anthrax immune globulin improves hemodynamics and survival during B. anthracis toxin-induced shock in canines receiving titrated fluid and vasopressor support
Source: Intensive Care Med Exp. 2017 Oct 23;5:48. doi: 10.1186/s40635-017-0159-9 (PMC5651533; doi:10.1186/s40635-017-0159-9)
Supplement: Supplementary file 5 — Differences in the effects of treatment at T2 or T5 versus T0 for arterial blood gas parameters. (DOCX 14 kb) [file 40635_2017_159_MOESM5_ESM.docx]

| Additional file 5: Table S5. Differences in the effects of treatment at T2 or T5 versus T0 for arterial blood gas parameters | | | | | | | | | |
| --- | --- | --- | --- | --- | --- | --- | --- | --- | --- |
| Parameter  (Unit) | Differences in the effect of treatment at  T2 versus T-4 (p-value) | | | |  | Differences in the effect of treatment at  T5 versus T-4 (p-value) | | | |
|  | Time of measurement | | | |  | Time of measurement | | | |
|  | 24 | 48 | 72 | 96 |  | 24 | 48 | 72 | 96 |
| Arterial pH | 0.03±0.03  (0.38) | 0.10±0.04  (0.04) | -0.01±0.10  (0.91) | 0.05±0.11  (0.70) |  | 0.003±0.04  (0.94) | 0.01±0.05  (0.92) | 0.10±0.12  (0.46) | - |
| Lactate  [Log_10_(mmol/L)] | -0.06±0.13  (0.63) | -0.31±0.19  (0.12) | -0.01±0.16  (0.96) | 0.07±0.10  (0.51) |  | 0.05±0.16  (0.77) | -0.19±0.22  (0.41) | -0.55±0.20  (0.02) | - |
| ABE  (mEq/L) | 0.10±1.5  (0.95) | 0.58±1.7  (0.74) | -1.7±3.3  (0.62) | -1.2±3.5  (0.75) |  | -3.1±1.8  (0.11) | -1.6±2.1  (0.47) | 6.3±4.2  (0.17) | - |
| PaO_2_  (mmHg) | -7±19  (0.72) | 12±32  (0.71) | -18±51  (0.73) | -3±51  (0.95) |  | 19±23  (0.42) | 2±39  (0.97) | 9±65  (0.90) | - |
| PaO_2_/FiO_2_ | -27±74  (0.72) | 48±127  (0.71) | 157±223  (0.50) | -84±220  (0.72) |  | 76±92  (0.42) | 6±158  (0.97) | -52±283  (0.86) | - |
| PaCO_2_  (mmHg) | -2±3  (0.49) | -7±3  (0.01) | -3±5  0.53) | -6±8  (0.47) |  | -5±4  (0.23) | -2±3  (0.50) | 13±6  (0.07) | - |
| ABE – arterial base excess; PaO_2_ – arterial oxygen pressure; PaO_2_/FiO_2_ - PaO_2_ to the fractional inspired oxygen concentration ratio; PaCO_2_ – arterial carbon dioxide pressure | | | | | | | | | |
