# Supplementary material for: Anthrax immune globulin improves hemodynamics and survival during B. anthracis toxin-induced shock in canines receiving titrated fluid and vasopressor support
Source: Intensive Care Med Exp. 2017 Oct 23;5:48. doi: 10.1186/s40635-017-0159-9 (PMC5651533; doi:10.1186/s40635-017-0159-9)
Supplement: Supplementary file 6 — Differences in the effects of treatment at T2 or T5 versus T0 for liver function parameters. (DOCX 14 kb) [file 40635_2017_159_MOESM6_ESM.docx]

| Additional file 6: Table S6. Differences in the effects of treatment at T2 or T5 versus T0 for liver function parameters | | | | | | | | | |
| --- | --- | --- | --- | --- | --- | --- | --- | --- | --- |
| Parameter  (Unit) | Differences in the effect of treatment at  T2 versus T-4 (p-value) | | | |  | Differences in the effect of treatment at  T5 versus T-4 (p-value) | | | |
|  | Time of measurement | | | |  | Time of measurement | | | |
|  | 24 | 48 | 72 | 96 |  | 24 | 48 | 72 | 96 |
| Total Bili  [Log_10_(U/L)] | 0.33±0.24  (0.19) | 0.02±0.25  (0.92) | 0.55±0.47  (0.28) | 0.11±0.42  (0.81) |  | 0.29±0.29  (0.33) | 0.22±0.30  (0.46) | 0.39±0.58  (0.52) | - |
| AST  [Log_10_(U/L)] | 0.55±0.16  (0.003) | 0.41±0.28  (0.12) | 1.3±0.40  (0.01) | 1.2±0.52  (0.06) |  | 0.44±0.19  (0.04) | 0.29±0.30  (0.34) | 0.18±0.49  (0.73) | - |
| ALT  [Log_10_(U/L)] | 0.16±0.22  (0.48) | -0.21±0.29  (0.50) | 0.96±0.52  (0.01) | 0.68±0.56  (0.27) |  | -0.13±0.27  (0.64) | -0.30±0.35  (0.40) | 0.002±0.65  (0.99) | - |
| LDH  [Log_10_(U/L)] | 0.35±0.21  (0.11) | -0.31±0.35  (0.40) | 0.46±0.43  (0.31) | 0.45±0.34  (0.23) |  | 0.52±0.26  (0.06) | 0.09±0.44  (0.84) | -0.18±0.53  (0.74) | - |
| Total Protein  (g/L) | 0.19±0.40  (0.65) | -0.31±0.75  (0.68) | 0.67±1.3  (0.96) | -0.12±0.27  (0.67) |  | 0.25±0.47  (0.60) | -0.80±0.93  (0.53) | 0.47±1.6  (0.77) | - |
| Albumin  (g/L) | 0.01±0.20  (0.98) | -0.31±0.26  (0.25) | 0.16±0.23  (0.52) | -0.14±0.21  (0.54) |  | 0.06±0.24  (0.81) | -0.14±0.32  (0.66) | -0.26±0.28  (0.38) | - |
| Total Bili – total bilirubin; AST – aspartate aminotransferase; ALT – alanine aminotransferase; LDH – lactate dehydrogenase | | | | | | | | | |
