# Supplementary material for: Anthrax immune globulin improves hemodynamics and survival during B. anthracis toxin-induced shock in canines receiving titrated fluid and vasopressor support
Source: Intensive Care Med Exp. 2017 Oct 23;5:48. doi: 10.1186/s40635-017-0159-9 (PMC5651533; doi:10.1186/s40635-017-0159-9)
Supplement: Supplementary file 7 — Differences in the effects of treatment at T2 or T5 versus T0 for platelets and coagulation parameters. (DOCX 13 kb) [file 40635_2017_159_MOESM7_ESM.docx]

| Additional file 7: Table S7. Differences in the effects of treatment at T2 or T5 versus T0 for platelets and coagulation parameters | | | | | | | | | |
| --- | --- | --- | --- | --- | --- | --- | --- | --- | --- |
| Parameter  (Unit) | Differences in the effect of treatment at  T2 versus T-4 (p-value) | | | |  | Differences in the effect of treatment at  T5 versus T-4 (p-value) | | | |
|  | Time of measurement | | | |  | Time of measurement | | | |
|  | 24 | 48 | 72 | 96 |  | 24 | 48 | 72 | 96 |
| Platelets  [Log_10_(x10^3^/L)] | -0.4±0.07  (0.60) | -0.17±0.13  (0.22) | 0.02±0.23  (0.92) | -0.57±0.16  (0.01) |  | 0.12±0.09  (0.18) | -0.29±0.17  (0.10) | -0.92±0.29  (0.01) | - |
| PT  (sec) | -2.3±1.6  (0.16) | -3.0±2.0  (0.15) | 3.0±3.3  (0.39) | -0.30±1.9  (0.88) |  | 3.2±1.9  (0.12) | 2.3±2.4  (0.35) | -8.4±4.0  (0.07) | - |
| aPTT  (sec) | 12.3±14.3  (0.40) | 13.8±30.0  (0.65) | 77±36  (0.06) | 12.3±53.8  (0.83) |  | -6.3±19.2  (0.75) | 55.3±36.9  (0.15) | - | - |
| PT – prothrombin time; aPTT – activated partial thromboplastin time | | | | | | | | | |
