# Supplementary material for: Anthrax immune globulin improves hemodynamics and survival during B. anthracis toxin-induced shock in canines receiving titrated fluid and vasopressor support
Source: Intensive Care Med Exp. 2017 Oct 23;5:48. doi: 10.1186/s40635-017-0159-9 (PMC5651533; doi:10.1186/s40635-017-0159-9)
Supplement: Supplementary file 8 — Mean (± SEM) values for parameters at baseline immediately before the start of toxin infusion in animals treated with anthrax immune globulin (AIG) or intravenous immune globulin (control) and the level of significance (p value) for the comparison of each parameter in the two groups. (DOCX 18 kb) [file 40635_2017_159_MOESM8_ESM.docx]

Additional file 8: Table S8. Mean (±SEM) values for parameters at baseline immediately before the start of toxin infusion in animals treated with anthrax immune globulin (AIG) or intravenous immune globulin (control) and the level of significance (p-value) for the comparison of each parameter in the two groups.

| **Parameter** | **Control**  **(mean ± SEM)** | **AIG**  **(mean ± SEM)** | **p- value** |
| --- | --- | --- | --- |
| Mean Arterial Blood Pressure (mmHg) | 91.80 ± 1.32 | 87.50 ± 2.72 | 0.17 |
| Shock score | 0.296 ± 0.06 | 0.100 ± 0.12 | 0.17 |
| Cardiac Index (mL/min/m^2^) | 133.90 ± 10.85 | 145.66 ± 12.43 | 0.48 |
| Central Venous Pressure (mmHg) | 5.27 ± 0.66 | 4.88 ± 0.52 | 0.64 |
| Heart Rate (bpm) | 68.60 ± 3.46 | 83.69 ± 5.77 | 0.03 |
| Left Ventricular Ejection Fraction (%) | 53.13 ± 1.00 | 53.38 ± 0.89 | 0.86 |
| Pulmonary Artery Diastolic Pressure (mmHg) | 10.33 ± 0.67 | 11.56 ± 1.02 | 0.33 |
| Pulmonary Artery Systolic Pressure (mmHg) | 25.60 ± 0.89 | 25.88 ± 1.35 | 0.87 |
| Mean Pulmonary Artery Pressure (mmHg) | 15.07 ± 0.65 | 16.31 ± 0.93 | 0.29 |
| Pulmonary Capillary Wedge Pressure (mmHg) | 7.27 ± 0.79 | 8.06 ± 0.69 | 0.45 |
| Pulmonary Vascular Resistance Index (dyn*s/cm^5^*m^2^) | 5.15 ± 0.65 | 5.13 ± 0.70 | 0.98 |
| Stroke Volume Index (mmHg/L/min) | 1.93 ± 0.09 | 1.78 ± 0.12 | 0.32 |
| Systemic Vascular Resistance Index (dyn*s/cm^5^*m^2^) | 54.44 ± 3.82 | 48.77 ± 4.90 | 0.37 |
| pH | 7.40 ± 0.01 | 7.41 ± 0.02 | 0.68 |
| Lactic Acid [log_10_(md/dL)] | 0.03 ± 0.03 | -0.003 ± 0.039 | 0.49 |
| Base Excess (mEq/L) | -8.26 ± 0.40 | -7.95 ±0.40 | 0.59 |
| PO_2_ (mmHg) | 174.08 ± 5.81 | 160.74 ± 9.53 | 0.25 |
| PCO_2_ (mmHg) | 26.78 ± 1.03 | 26.67 ± 1.18 | 0.94 |
| PaO_2_:FiO_2_ | 696.34 ± 23.25 | 642.97 ± 38.10 | 0.25 |
| Sodium (mEq/L) | 142.06 ± 4.65 | 148.97 ± 1.72 | 0.18 |
| Potassium (mEq/L) | 3.72 ± 0.17 | 4.02 ± 0.10 | 0.13 |
| Chloride (mEq/L) | 118.24 ± 3.94 | 123.85 ± 1.94 | 0.22 |
| Bicarbonate (mEq/L) | 16.79 ± 0.36 | 17.00 ± 0.41 | 0.71 |
| BUN [log_10_(mg/dL)] | 0.94 ± 0.04 | 0.99 ± 0.03 | 0.34 |
| Creatinine [log_10_(mg/dL)] | -0.35 ± 0.03 | -0.31 ± 0.02 | 0.36 |
| Glucose (mg/dL) | 115.7 ± 9.0 | 112.4 ± 9.4 | 0.80 |
| BUN:Creatinine | 19.67 ± 0.78 | 20.41 ± 1.32 | 0.64 |
| Calcium (mg/dL) | 1.22 ± 0.02 | 1.20 ± 0.02 | 0.53 |
| ALT [log_10_(U/L)] | 1.25 ± 0.05 | 1.30 ± 0.04 | 0.47 |
| AST [log_10_(U/L)] | 1.41 ± 0.06 | 1.44 ± 0.05 | 0.68 |
| Total Bilirubin [log_10_(mmol/L)] | -0.77 ± 0.07 | -0.69 ± 0.06 | 0.40 |
| Albumin (g/dL) | 2.10 ± 0.12 | 2.21 ± 0.09 | 0.48 |
| LDH [log_10_(U/L)] | 1.67 ± 0.04 | 1.81 ± 0.05 | 0.06 |
| White Blood Cell Count (x10^3^) | 5.58 ± 0.87 | 4.66 ± 0.44 | 0.27 |
| Lymphocyte count [log_10_(x10^3^)] | 0.19 ± 0.08 | 0.24 ± 0.06 | 0.61 |
| Neutrophil (x10^3^) | 3.56 ± 0.73 | 2.05 ± 0.42 | 0.07 |
| Hemoglobin (g/dL) | 10.57 ± 0.95 | 10.71 ± 1.05 | 0.92 |
| Platelet count [log 10] (x10^3^) | 2.48 ± 0.05 | 2.51 ± 0.06 | 0.69 |
| PT (sec) | 12.60 ± 0.41 | 12.75 ± 0.43 | 0.80 |
| aPTT (sec) | 81.20 ± 2.21 | 86.50 ± 2.29 | 0.11 |
