# Supplementary figures and images for: Anthrax immune globulin improves hemodynamics and survival during B. anthracis toxin-induced shock in canines receiving titrated fluid and vasopressor support
Source: Intensive Care Med Exp. 2017 Oct 23;5:48. doi: 10.1186/s40635-017-0159-9 (PMC5651533; doi:10.1186/s40635-017-0159-9)

Supplementary Figure 1

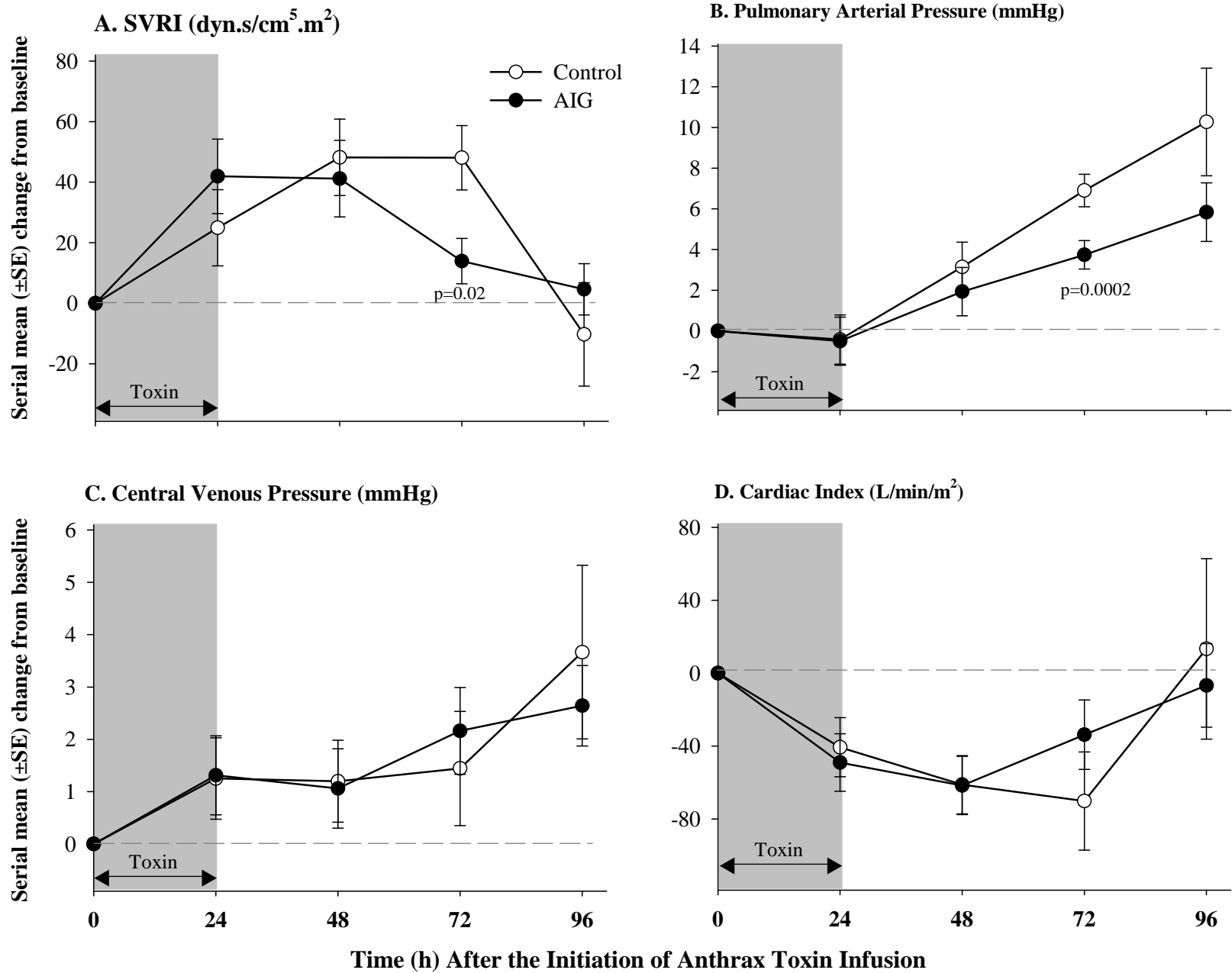

Supplement: Supplementary file 9 — Serial mean (± SEM) changes from baseline in systeic vascular resistance index (a), pulmonary arterial pressure (b), central venous pressure (c) and cardiac index (d) comparing animals receiving anthrax immune globulin (AIG, black circles) versus intravenous immune globulin (control, open circles). Levels of significance (p values) for time points at which the groups differed significantly are provided in the figure. (PDF 14 kb) [file 40635_2017_159_MOESM9_ESM.pdf]
